# Supplementary material for: Epidemiology of type 2 diabetes remission in Scotland in 2019: A cross-sectional population-based study
Source: PLoS Med. 2021 Nov 2;18(11):e1003828. doi: 10.1371/journal.pmed.1003828 (PMC8562803; doi:10.1371/journal.pmed.1003828)
Supplement: S2 Table — Number of procedures for people with an obesity code are given against each OPCS code for the present analysis. ICD-10, International Classification of Diseases-10th revision; OPCS-4, Office of Population Censuses and Surveys Classification of Interventions and Procedures version 4. (DOCX) [file pmed.1003828.s002.docx]

S2 Table: OPCS-4 procedure codes used in combination with ICD-10 diagnosis codes for obesity and type 2 diabetes by Public Health Scotland to identify people with a history of bariatric surgery. Number of procedures for people with an obesity code are given against each OPCS code for the present analysis.

| General bariatric procedure | OPCS code included | Included codes | Number |
| --- | --- | --- | --- |
| G27: Total excision of stomach | G27.1 | Total gastrectomy and excision of surrounding tissue | 0 |
|  | G27.2 | Total gastrectomy and anastomosis of oesophagus to duodenum | 3 |
|  | G27.3 | Total gastrectomy and interposition of jejunum | 0 |
|  | G27.4 | Total gastrectomy and anastomosis of oesophagus to transposed jejunum | 0 |
|  | G27.5 | Total gastrectomy and anastomosis of oesophagus to jejunum NEC | 3 |
|  | G27.8 | Other specified | 0 |
|  | G27.9 | Unspecified | 4 |
| G 28: Partial excision of stomach | G28.1 | Partial gastrectomy and anastomosis of stomach to duodenum | 1 |
|  | G28.2 | Partial gastrectomy and anastomosis of stomach to transposed jejunum | 5 |
|  | G28.3 | Partial gastrectomy and anastomosis of stomach to jejunum NEC | 5 |
|  | G28.4 | Sleeve gastrectomy and duodenal switch  Excludes: Duodenal switch (G71.6) | 1 |
|  | G28.5 | Sleeve gastrectomy NEC | 136 |
|  | G28.9 | Unspecified. Includes: Gastrectomy NEC | 7 |
| G 30: Plastic operations on stomach | G30.1 | Gastroplasty NEC | 33 |
|  | G30.3 | Partitioning of stomach using band | 118 |
|  | G30.4 | Partitioning of stomach using staples | 15 |
|  | G30.5 | Maintenance of gastric band NCCS. Excludes: Removal of gastric band ([G38.7](https://classbrowser.nhs.uk/OPCS-4.9/volume1-p2-3.html#G38.7)) | 13 |
|  | G30.9 | Unspecified | 3 |
| G 31: Connection of stomach to duodenum | G31.0 | Conversion from previous anastomosis of stomach to duodenum | 0 |
|  | G31.1 | Bypass of stomach by anastomosis of oesophagus to duodenum | 1 |
|  | G31.2 | Bypass of stomach by anastomosis of stomach to duodenum | 1 |
|  | G31.3 | Revision of anastomosis of stomach to duodenum | 0 |
|  | G31.4 | Conversion to anastomosis of stomach to duodenum | 0 |
|  | G31.5 | Closure of connection of stomach to duodenum | 0 |
|  | G31.6 | Attention to connection of stomach to duodenum  Excludes: Revision of anastomosis of stomach to duodenum (G31.3)  Closure of connection of stomach to duodenum (G31.5) | 0 |
|  | G31.8 | Other specified | 0 |
|  | G31.9 | Unspecified | 1 |
| G32: Connection of stomach to transposed jejunum | G32.0 | Conversion from previous anastomosis of stomach to transposed jejunum | 0 |
|  | G32.1 | Bypass of stomach by anastomosis of stomach to transposed jejunum | 33 |
|  | G32.2 | Revision of anastomosis of stomach to transposed jejunum | 0 |
|  | G32.3 | Conversion to anastomosis of stomach to transposed jejunum | 0 |
|  | G32.4 | Closure of connection of stomach to transposed jejunum | 0 |
|  | G32.5 | Attention to connection of stomach to transposed jejunum | 0 |
|  | G32.8 | Other specified | 0 |
|  | G32.9 | Unspecified | 4 |
| G33: Other connection of stomach to jejunum | G33.0 | Conversion from previous anastomosis of stomach to jejunum NEC |  |
|  | G33.1 | Bypass of stomach by anastomosis of stomach to jejunum NEC | 83 |
|  | G33.2 | Revision of anastomosis of stomach to jejunum NEC | 1 |
|  | G33.3 | Conversion to anastomosis of stomach to jejunum NEC | 0 |
|  | G33.4 | Open reduction of intussusception of gastroenterostomy | 0 |
|  | G33.5 | Closure of connection of stomach to jejunum NEC | 0 |
|  | G33.6 | Attention to connection of stomach to jejunum  -Excludes: Revision of anastomosis of stomach to jejunum NEC ([G33.2](https://classbrowser.nhs.uk/OPCS-4.9/volume1-p2-3.html#G33.2)) Closure of anastomosis of stomach to jejunum NEC ([G33.5](https://classbrowser.nhs.uk/OPCS-4.9/volume1-p2-3.html#G33.5)) | 1 |
|  | G33.8 | Other specified | 1 |
|  | G33.9 | Unspecified | 19 |
| G38 Other open operations on stomach | G38.7 | Removal of gastric band | 0 |
| G48: Other operations on stomach | G48.1 | Insertion of gastric bubble | 17 |
|  | G48.2 | Attention to gastric bubble | 71 |
|  | G48.5 | Insertion of gastric balloon | 20 |
|  | G48.6 | Attention to gastric balloon  NCCS 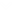 | 8 |
| G71: Bypass of ileum | G71.6 | Duodenal switch | 0 |
|  | G71.7 | Reversal of duodenal switch | 0 |
