# Supplementary material for: Epidemiology of type 2 diabetes remission in Scotland in 2019: A cross-sectional population-based study
Source: PLoS Med. 2021 Nov 2;18(11):e1003828. doi: 10.1371/journal.pmed.1003828 (PMC8562803; doi:10.1371/journal.pmed.1003828)
Supplement: S3 Table — ICD-9, International Classification of Diseases-Ninth revision; ICD-10, International Classification of Diseases-10th revision. (DOCX) [file pmed.1003828.s003.docx]

S3 Table ICD-10 and ICD-9 codes used to identify comorbidities

| Comorbidity | ICD-10 code | ICD-9 code |
| --- | --- | --- |
| Dementia | F00, F01, F02, F03, F051 | -290, -2941 |
| Chronic kidney disease 5 | N185, N19 | -585.5, -585.6 |
| Liver cirrhosis | K74, K717 | -5712, -5715 |
| Metastatic cancer in the last 5 years | C77-79 | -196-198 |
| Cancer (excluding non-melanoma skin cancers) in the last 5 years | C0-43, 45, 97 | -140-172, 174-208 |
