# Supplementary material for: Epidemiology of type 2 diabetes remission in Scotland in 2019: A cross-sectional population-based study
Source: PLoS Med. 2021 Nov 2;18(11):e1003828. doi: 10.1371/journal.pmed.1003828 (PMC8562803; doi:10.1371/journal.pmed.1003828)
Supplement: S4 Table — Odds of missing data in people with type 2 diabetes in Scotland diagnosed ≥30 years of age who had at least 1 HbA1c ≥48 mmol/mol (6.5%) after diagnosis of diabetes and who were alive and had at least 1 HbA1c recorded in 2019. (DOCX) [file pmed.1003828.s004.docx]

S4 Table Proportions of missing data within the variable of interest. Odds of missing data in people with type 2 diabetes in Scotland diagnosed >30 years of age who had at least one HbA1c >48mmol/mol (6.5%) after diagnosis of diabetes and who were alive and had at least one HbA1c recorded in 2019

|  | Complete | Missing | Unadjusted OR | P |
| --- | --- | --- | --- | --- |
| **Remission status** |  |  |  |  |
| No remission (ref) | 111411 (71.2) | 43195 (27.9) | - |  |
| Remission | 5637 (73.1) | 2073 (26.9) | 0.95 (0.90-1.00) | 0.045 |
| **Age in 2019 (years)** |  |  |  |  |
| Mean (SD) | 66.5 (11.5) | 66.6 (12.4) | 1 (1.00-1.00) | 0.03 |
| **Sex** |  |  |  |  |
| Female (ref) | 49375 (71.0) | 20153 (29.0) |  |  |
| Male | 67673 (72.9) | 25115 (21.1) | 0.91 (0.89-0.93) | <0.001 |
| **HbA1c at diagnosis (mmol/mol)** |  |  |  |  |
| Mean (SD) | 60.8 (17.7) | 60.9 (18.3) | 1.00 (1.00-1.00) | 0.497 |
| **Weight change from diagnosis to 2019 (kg)** |  |  |  |  |
| Mean (SD) | -4.3 (8.6) | -4.4 (10.2) | 1.00 (1.00-1.00) | 0.188 |
| **Current or previous history of GLT** |  |  |  |  |
| GLT therapy | 95160 (72.5) | 36163 (27.5) |  |  |
| no previous therapy | 21888 (70.6) | 9105 (29.4) | 0.91 (0.89-0.94) | <0.001 |
| **Previous history of bariatric surgery** |  |  |  |  |
| Bariatric surgery | 326 (66.8) | 162 (33.2) |  |  |
| No previous bariatric surgery | 116722 (72.1) | 45106 (27.9) | 0.78(0.65-0.94) | 0.009 |
| **Past Medical History** |  |  |  |  |
| No history of dementia | 116453 (72.3) | 44581 (27.7) |  |  |
| Dementia | 595 (46.4) | 687 (53.6) | 3.02 (2.70-3.37) | <0.001 |
| No history of liver cirrhosis | 116202 (72.1) | 44871 (27.9) |  |  |
| Liver cirrhosis | 846 (68.1) | 397 (31.9) | 1.22(1.08-1.37) | P=0.001 |
| No history of end stage kidney disease | 116357 (72.2) | 44905 (27.8) |  |  |
| end stage kidney disease | 691 (65.6) | 363 (34.4) | 1.36(1.20-1.55) | <0.001 |
| No history of cancer | 112728 (72.2) | 43414 (27.8) |  |  |
| Cancer | 4320 (70.0) | 1854 (30.0) | 1.11(1.05-1.18) | p<0.001 |
| No metastases | 116284 (72.1) | 44939 (27.9) |  |  |
| Metastases | 764 (69.9) | 329 (30.1) | 1.11(0.98-1.27) | 0.102 |
