# Supplementary material for: Epidemiology of type 2 diabetes remission in Scotland in 2019: A cross-sectional population-based study
Source: PLoS Med. 2021 Nov 2;18(11):e1003828. doi: 10.1371/journal.pmed.1003828 (PMC8562803; doi:10.1371/journal.pmed.1003828)
Supplement: S5 Table — Adjusted for all variables in the model (listed in first column of the table). CCA, Complete case analysis. (DOCX) [file pmed.1003828.s005.docx]

S5 Table. Unadjusted and adjusted odds for remission of type 2 diabetes from logistic regression model using complete case and multiple imputation datasets ^1^ N= 162,316 (Multiple imputation) N=117,048 (CCA) Adjusted for all variables in the model (listed in first column of the table).

|  | No remission | Remission | CCA unadjusted OR | P | CCA adjusted OR | P | MI unadjusted OR | P | MI adjusted OR ^1^ | P |
| --- | --- | --- | --- | --- | --- | --- | --- | --- | --- | --- |
| Age in 2019 (yrs) |  |  |  |  |  |  |  |  |  |  |
| >30 to <45 | 5977 (96.8) | 200 (3.2) | 1.08 (0.91-1.26) | 0.34 | 1.13 (0.92-1.39) | 0.24 | 1.08 (0.91-1.26) | 0.37 | 1.16 (0.98 -1.38) | 0.09 |
| >45 to <55 | 2124 (97.0) | 666 (3.0) | - | - | - | - | - | - | - | - |
| >55 to <65 | 42208 (96.7) | 1430 (3.3) | 1.09 (0.99-1.20) | 0.07 | 1.02 (0.90-1.15) | 0.78 | 1.09 (0.99- 1.20) | 0.07 | 0.99 (0.89-1.09) | 0.74 |
| >65 to <75 | 47515 (95.3) | 2342 (4.7) | 1.59 (1.45-1.73) | <0.001 | 1.15 (1.02-1.28) | 0.02 | 1.59 (1.45-1.73) | <0.001 | 1.20 (1.09-1.32) | <0.001 |
| >75 | 37482 (92.4) | 3072 (7.6) | 2.64 (2.42-2.87) | <0.001 | 1.35 (1.21-1.52) | <0.001 | 2.64 (2.42- 2.87) | <0.001 | 1.48 (1.34-1.62) | <0.001 |
| Sex |  |  |  |  |  |  |  |  |  |  |
| Female | 65942 (94.8) | 3586 (5.2) | - | - | - | - | - | - | - | - |
| Male | 88664 (95.6) | 4124 (4.4) | 0.86 (0.82-0.90) | <0.001 | 1.10 (1.03-1.16) | 0.002 | 0.86 (0.82- 0.90) | <0.001 | 1.03 (0.98-1.08) | 0.25 |
| HbA1c at diagnosis mmol/mol |  |  |  |  |  |  |  |  |  |  |
| <48 | 25558 (90.2) | 2773 (9.8) | 1.32 (1.25-1.39) | <0.001 | 1.37 (1.27-1.46) | <0.001 | 1.30 (1.23-1.37) | <0.001 | 1.31 (1.24-1.39) | <0.001 |
| 48 to 52.9 | 32211 (92.4) | 2655 (7.6) |  |  |  |  |  |  |  |  |
| 53 to 63.9 | 34984 (96.7) | 1193 (3.3) | 0.41 (0.39-0.44) | <0.001 | 0.72 (0.66-0.79) | <0.001 | 0.42 (0.39-0.45) | <0.001 | 0.74 (0.68-0.80) | <0.001 |
| 64 to 85.9 | 29973 (98.3) | 514 (1.7) | 0.21 (0.19-0.23) | <0.001 | 0.75 (0.66-0.85) | <0.001 | 0.21 (0.19-0.23) | <0.001 | 0.72 (0.65-0.80) | <0.001 |
| 86+ | 15758 (99.2) | 130 (0.8) | 0.10 (0.08-0.12) | <0.001 | 0.50 (0.40-0.61) | <0.001 | 0.11 (0.09-0.13) | <0.001 | 0.47 (0.39-0.57) | <0.001 |
| Weight change(kg) |  |  |  |  |  |  |  |  |  |  |
| >5.0 gain | 11719 (98.1) | 227 (1.9) | 0.64 (0.55-0.74) | <0.001 | 0.97 (0.82-1.14) | 0.70 | 0.66 (0.56-0.79) | <0.001 | 0.91 (0.76-1.09) | 0.31 |
| 0 to 4.9 gain | 23530 (97.0) | 717 (3.0) | - | - | - | - | - | - | - | - |
| 0.1 to 4.9 loss | 34265 (96.0) | 1439 (4.0) | 1.38 (1.26-1.51) | <0.001 | 1.42 (1.29-1.57) | <0.001 | 1.10 (0.97-1.23) | 0.13 | 1.12 (0.99-1.27) | 0.066 |
| 5 to 9.9 loss | 25401 (94.6) | 1458 (5.4) | 1.88 (1.72-2.06) | <0.001 | 2.74 (2.48-3.03) | <0.001 | 1.52 (1.35-1.71) | <0.001 | 2.11 (1.86-2.40) | <0.001 |
| 10 to 14.9 loss | 13006 (93.6) | 894 (6.4) | 2.26 (2.04-2.49) | <0.001 | 4.30 (3.84-4.81) | <0.001 | 1.73 (1.53-1.96) | <0.001 | 2.95 (2.57-3.39) | <0.001 |
| >15kg loss | 10397 (90.3) | 1123 (9.7) | 3.54 (3.22-3.91) | <0.001 | 8.33 (7.44-9.33) | <0.001 | 2.42 (2.15-2.73) | <0.001 | 4.45 (3.89-5.10) | <0.001 |
| GLT |  |  |  |  |  |  |  |  |  |  |
| Previous GLT | 129389 (98.5) | 1934 (1.5) |  |  |  |  |  |  |  |  |
| No GLT | 25217 (81.4) | 5776 (18.6) | 15.32 (14.53-16.17) | <0.001 | 19.34 (17.94-20.88) | <0.001 | 15.3(14.5-16.2) | <0.001 | 14.57 (13.66-15.54) | <0.001 |
| History of bariatric surgery |  |  |  |  |  |  |  |  |  |  |
| Bariatric surgery | 362 (74.2) | 126 (25.8) |  |  |  |  |  |  |  |  |
| No bariatric | 154244 (95.3) | 7584 (4.7) | 7.08 (5.75-8.65) | <0.001 | 9.78 (7.32-12.94) | <0.001 | 7.08 (5.77-8.68) | <0.001 | 11.93 (9.41-15.13) | <0.001 |
| Duration of diabetes (years) |  |  |  |  |  |  |  |  |  |  |
| **≤**2 | 10335 (97.9) | 224 (2.1) | 0.30 (0.26-0.34) | <0.001 |  |  | 0.22 (0.19-0.25) | <0.001 |  |  |
| 2.1-6 | 45113 (93.2) | 3300 (6.8) | - | - |  |  | - | - |  |  |
| >6 | 99158 (95.9) | 4186 (4.1) | 0.58 (0.55-0.60) | <0.001 |  |  | 0.85 (0.80-0.90) | <0.001 |  |  |
| Deprivation |  |  |  |  |  |  |  |  |  |  |
| SIMD 2016 quintile 1 | 36352 (95.8) | 1594 (4.2) |  |  |  |  |  |  |  |  |
| 2 | 35550 (95.5) | 1668 (4.5) | 1.07 (1.00-1.15) | 0.059 |  |  | 1.07 (1.00-1.15) | 0.059 |  |  |
| 3 | 31473 (95.1) | 1631 (4.9) | 1.18 (1.10-1.27) | <0.001 |  |  | 1.18 (1.10-1.27) | <0.001 |  |  |
| 4 | 27744 (95.0) | 1449 (5.0) | 1.19 (1.11-1.28) | <0.001 |  |  | 1.19 (1.11-1.28) | <0.001 |  |  |
| 5 | 21883 (94.3) | 1312 (5.7) | 1.37 (1.27-1.47) | <0.001 |  |  | 1.37 (1.27-1.48) | <0.001 |  |  |
| Never Smoker | 71361 (95.2) | 3571 (4.8) |  |  |  |  |  |  |  |  |
| Ex-smoker | 51844 (94.6) | 2963 (5.4) | 1.14 (1.09-1.20) | <0.001 |  |  | 1.14 (1.09-1.20) | <0.001 |  |  |
| Current Smoker | 31214 (96.4) | 1170 (3.6) | 0.75 (0.70-0.80) | <0.001 |  |  | 0.75 (0.70-0.80) | <0.001 |  |  |
