# Supplementary material for: Epidemiology of type 2 diabetes remission in Scotland in 2019: A cross-sectional population-based study
Source: PLoS Med. 2021 Nov 2;18(11):e1003828. doi: 10.1371/journal.pmed.1003828 (PMC8562803; doi:10.1371/journal.pmed.1003828)
Supplement: S6 Table — Unadjusted and adjusted odds for remission of type 2 diabetes from logistic regression model using complete case and multiple imputation datasets. 1 Adjusted for all variables in the model (listed in first column of the table). N = 152 934 (multiple imputation) and N = 110,814 (CCA). CCA, complete case analysis. (DOCX) [file pmed.1003828.s006.docx]

S6 Table Sensitivity Analysis with 10 846 people with comorbidities removed. Unadjusted and adjusted odds for remission of type 2 diabetes from logistic regression model using complete case and multiple imputation datasets ^1^ Adjusted for all variables in the model (listed in first column of the table). N=152 934 (Multiple imputation) N=110,814 (CCA)

|  | No remission | Remission | CCA unadjusted OR | P | CCA adjusted OR | P | MI unadjusted OR | P | MI adjusted OR | P |
| --- | --- | --- | --- | --- | --- | --- | --- | --- | --- | --- |
| **Age in 2019 (yrs)** |  |  |  |  |  |  |  |  |  |  |
|  | 5903 (96.8) | 197 (3.2) | 1.09 (0.92-1.28) | 0.30 | 1.14 (0.92-1.40) | 0.22 | 1.09 (0.93-1.28) | 0.30 | 1.18(0.99-1.40) | 0.07 |
| 45 to 54 | 20982 (97.0) | 643 (3.0) | - | - | - | - | - | - | - | - |
| 55 to 65 | 40577 (96.8) | 1353 (3.2) | 1.09 (0.99-1.20) | 0.08 | 1.02 (0.90-1.15) | 0.74 | 1.09 (0.99-1.20) | 0.08 | 0.98 (0.89-1.09) | 0.75 |
| 65 to 75 | 44429 (95.4) | 2146 (4.6) | 1.58 (1.44-1.73) | <0.001 | 1.15 (1.03-1.29) | 0.018 | 1.58 (1.44-1.72) | <0.001 | 1.19 (1.08-1.31) | <0.001 |
| 75+ | 33947 (92.5) | 2757 (7.5) | 2.65 (2.43-2.89) | <0.001 | 1.36 (1.21-1.53) | <0.001 | 2.65 (2.43-2.89) | <0.001 | 1.46 (1.33-1.61) | <0.001 |
| **Sex** |  |  |  |  |  |  |  |  |  |  |
| Female | 61972 (95.0) | 3291 (5.0) | - | - | - | - | - | - | - | - |
| Male | 83866 (95.7) | 3805 (4.3) | 0.85 (0.81-0.90) | <0.001 | 1.09 (1.03-1.16) | 0.004 | 0.85 (0.81- 0.90) | <0.001 | 1.04 (0.98-1.09) | 0.19 |
| **Diagnosis HbA1c mmol/mol** |  |  |  |  |  |  |  |  |  |  |
| <48 | 23981 (90.4) | 2554 (9.6) | 1.31 (1.24-1.39) | <0.001 | 1.37 (1.27-1.47) | <0.001 | 1.30 (1.23-1.37) | <0.001 | 1.32 (1.24-1.40) | <0.001 |
| 48 to 52.9 | 30278 (92.5) | 2456 (7.5) |  |  |  |  |  |  |  |  |
| 53 to 63.9 | 32997 (96.8) | 1095 (3.2) | 0.41 (0.38-0.44) | <0.001 | 0.72 (0.65-0.78) | <0.001 | 0.41 (0.38-0.44) | <0.001 | 0.74 (0.68-0.80) | <0.001 |
| 64 to 85.9 | 28368 (98.4) | 465 (1.6) | 0.20 (0.18-0.44) | <0.001 | 0.75 (0.66-0.86) | <0.001 | 0.21 (0.19-0.23) | <0.001 | 0.72 (0.64-0.80) | <0.001 |
| 86+ | 14970 (99.2) | 114 (0.8) | 0.09 (0.08-0.11) | <0.001 | 0.49 (0.39-0.62) | <0.001 | 0.10 (0.08-0.12) | <0.001 | 0.46 (0.38-0.56) | <0.001 |
| **Weight change (kg)** |  |  |  |  |  |  |  |  |  |  |
| 5+ gain | 11088 (98.1) | 211 (1.9) | 0.63 (0.53-0.73) | <0.001 | 0.95 (0.81-1.13) | 0.58 | 0.64 (0.54-0.76) | <0.001 | 0.88 (0.73-1.07) | 0.19 |
| 0 to 4.9 gain | 22473 (97.0) | 684 (3.0) | - | - | - | - | - | - | - | - |
| 0.1 to 4.9 loss | 32722 (96.0) | 1374 (4.0) | 1.38 (1.26-1.52) | <0.001 | 1.43 (1.29-1.58) | <0.001 | 1.11 (0.98-1.25) | 0.09 | 1.14 (1.01-1.30) | 0.04 |
| 5 to 9.9 loss | 24080 (94.6) | 1364 (5.4) | 1.86 (1.70-2.04) | <0.001 | 2.74 (2.47-3.03) | <0.001 | 1.52 (1.35-1.71) | <0.001 | 2.14 (1.88-2.44) | <0.001 |
| 10 to 14.9 loss | 12190 (93.7) | 814 (6.3) | 2.19 (1.98-2.43) | <0.001 | 4.31 (3.83-4.84) | <0.001 | 1.71 (1.51-1.94) | <0.001 | 2.99 (2.59-3.45) | <0.001 |
| 15+ loss | 9546 (90.5) | 1002 (9.5) | 3.45 (3.12-3.81) | <0.001 | 8.33 (7.41-9.38) | <0.001 | 2.39 (2.12-2.70) | <0.001 | 4.48 (3.90-5.14) | <0.001 |
| **GLT** |  |  |  |  |  |  |  |  |  |  |
| Previous GLT | 121966 (98.6) | 1716 (1.4) |  |  |  |  |  |  |  |  |
| No GLT | 23872 (81.6) | 5380 (18.4) | 16.02 (15.15-16.95) | <0.001 | 20.16 (18.63-21.84) | <0.001 | 16.02 (15.14- 16.94) | <0.001 | 15.29 (14.29-16.35) | <0.001 |
| **Bariatric surgery** |  |  |  |  |  |  |  |  |  |  |
| No Previous | 145502 (95.4) | 6978 (4.6) |  |  |  |  |  |  |  |  |
| Previous surgery | 336 (74.0) | 118 (26.0) | 7.32 (5.91-9.01) | <0.001 | 10.39 (7.68-13.90) | <0.001 | 7.32 (5.93-9.04) | <0.001 | 13.01 (10.17-16.64) | <0.001 |
