# Supplementary material for: Epidemiology of type 2 diabetes remission in Scotland in 2019: A cross-sectional population-based study
Source: PLoS Med. 2021 Nov 2;18(11):e1003828. doi: 10.1371/journal.pmed.1003828 (PMC8562803; doi:10.1371/journal.pmed.1003828)

S2 Fig Prevalence of remission of type 2 diabetes amongst the Scottish type 2 diabetes population of people who had at least one HbA1c>48mmol/mol (6.5%) after diagnosis of diabetes and had at least one HbA1c recorded in 2019 by age in 2019.


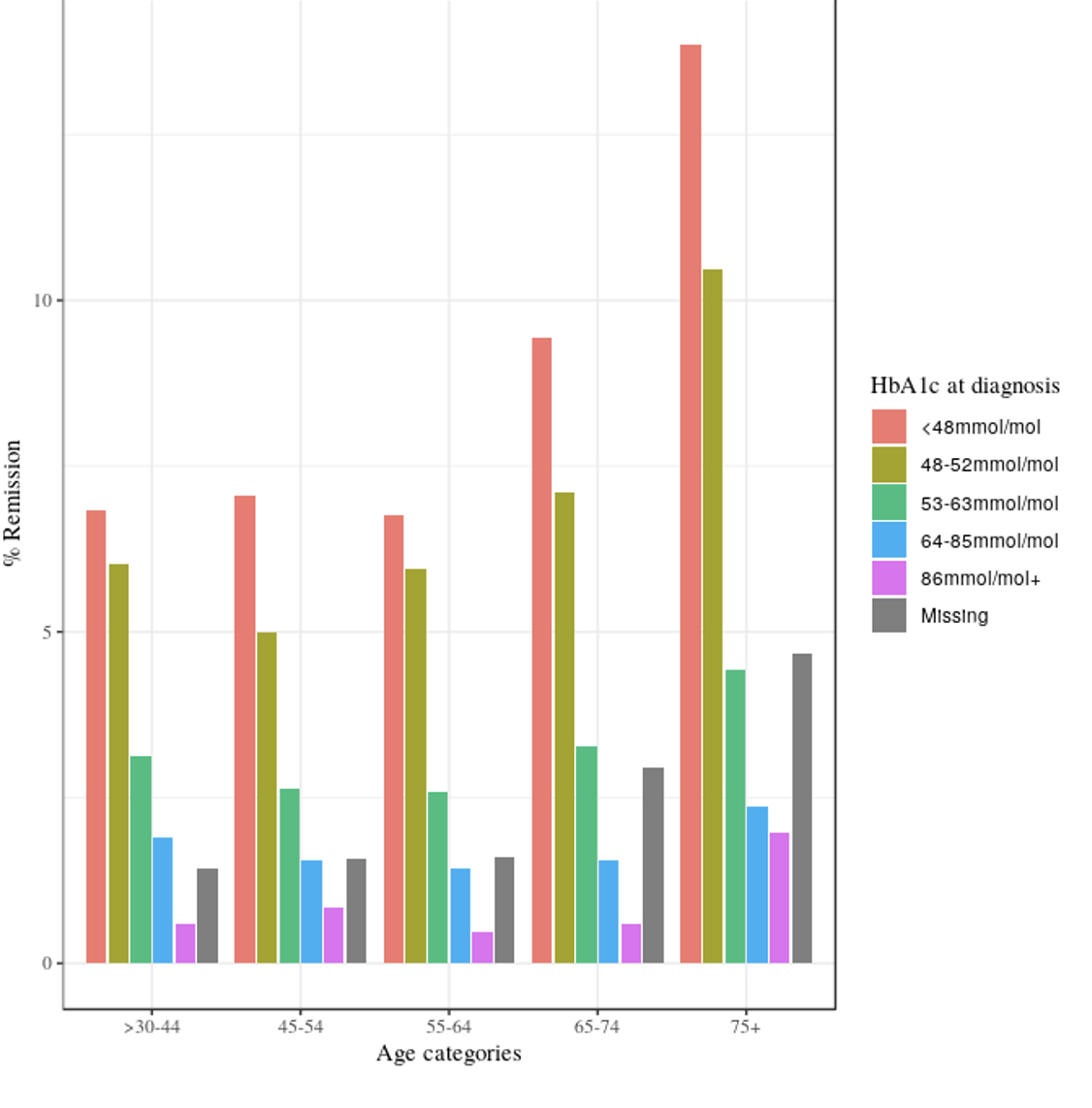

Supplement: S2 Fig — (DOCX) [file pmed.1003828.s008.docx]
