# Supplementary material for: Epidemiology of type 2 diabetes remission in Scotland in 2019: A cross-sectional population-based study
Source: PLoS Med. 2021 Nov 2;18(11):e1003828. doi: 10.1371/journal.pmed.1003828 (PMC8562803; doi:10.1371/journal.pmed.1003828)

S3 Fig: Median weight change (kg) from diagnosis of type 2 diabetes to 2019 according to remission (n=7710) and non-remission status (n=154 606)


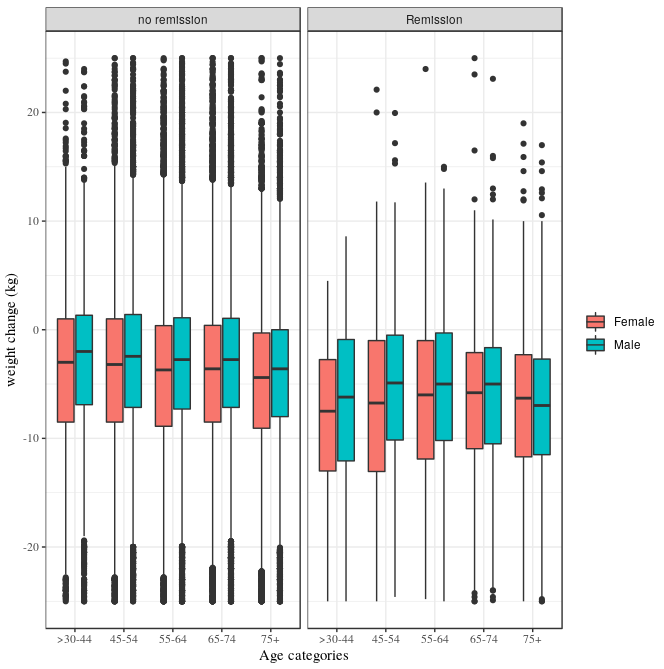

Supplement: S3 Fig — (DOCX) [file pmed.1003828.s009.docx]
