# Supplementary material for: Epidemiology of type 2 diabetes remission in Scotland in 2019: A cross-sectional population-based study
Source: PLoS Med. 2021 Nov 2;18(11):e1003828. doi: 10.1371/journal.pmed.1003828 (PMC8562803; doi:10.1371/journal.pmed.1003828)
Supplement: S4 Fig — N = 117,048. CI, confidence interval; OR, odds ratio. (DOCX) [file pmed.1003828.s010.docx]

S4 Fig. Odds ratios for remission of type 2 diabetes (95% CI) in Scotland 2019 derived from complete case analysis logistic regression model adjusted for all covariables listed on the plot. N=117,048
