# Supplementary material for: Epidemiology of type 2 diabetes remission in Scotland in 2019: A cross-sectional population-based study
Source: PLoS Med. 2021 Nov 2;18(11):e1003828. doi: 10.1371/journal.pmed.1003828 (PMC8562803; doi:10.1371/journal.pmed.1003828)
Supplement: S5 Fig — People with previous history of dementia, end-stage renal disease, liver cirrhosis, cancer, or metastases in the last 5 years removed. N = 110,814. CI, confidence interval; OR, odds ratio. (DOCX) [file pmed.1003828.s011.docx]

S5 Fig: Odds ratios for remission of type 2 diabetes (95% CI) in Scotland 2019 derived from complete case analysis logistic regression model adjusted for all covariables listed on the plot. People with previous history of dementia, end-stage renal disease, liver cirrhosis, cancer or metastases in the last 5 years removed. N=110814
