# Supplementary material for: Epidemiology of type 2 diabetes remission in Scotland in 2019: A cross-sectional population-based study
Source: PLoS Med. 2021 Nov 2;18(11):e1003828. doi: 10.1371/journal.pmed.1003828 (PMC8562803; doi:10.1371/journal.pmed.1003828)
Supplement: Attachment — Submitted filename: Response to reviewers.pdf [file pmed.1003828.s012.pdf]

THE UNIVERSITY of EDINBURGH | usher institute

NINE Edinburgh BioQuarter
9 Little France Road
Edinburgh EH16 4UX
usher.bioquarter@ed.ac.uk

Old Medical School
Teviot Place
Edinburgh EH8 9AG
usher.teviot@ed.ac.uk

www.ed.ac.uk/usher

Dr Richard Turner
Senior Editor
PLOS Medicine

4th September 2021

Dear Dr Turner

Re: Epidemiology of type 2 diabetes remission in Scotland in 2019: a cross-sectional population-based study

We thank all the reviewers for their time and attention. We have attached the reviewers' comments and our responses below.

Yours sincerely,

Dr Mireille Captieux, on behalf of all authors.

Response to editors' comments:

Table 1 Academic Editor

Point	Comment	Action/Response
	Requests	
1	At line 37, please add "We carried out a ..." or similar.	We have modified this comment in paragraph 2 of the abstract.
2	In the abstract, please quote aggregate demographic details for study participants.	We have added aggregate demographic details in paragraph 2 of the abstract.
3	Please add a new final sentence to the "Methods and findings" subsection of your abstract, beginning "Study limitations include ..." or similar and quoting 2-3 of the study's main limitations.	We have added a section on limitations at the end of paragraph 2 of the abstract
4	At line 55, please begin the sentence with "In this study, we found that ..." or similar.	We have changed the beginning of the sentence as suggested in paragraph 3 of the abstract.
5	Again at line 55, please avoid statements of the type "almost X" in favour of quoting the actual number or proportion.	We have quoted the actual proportion as suggested in paragraph 3 of the abstract.
6	Please remove the information on funding from the abstract page. In the event of publication, this information will be included in the article metadata, via entries in the submission form.	We have removed funding information from the abstract page.
7	After the abstract, we will need to ask you to add a new and accessible "Author summary" section in non-identical prose. You may find it helpful to consult one or two recent research papers published in PLOS Medicine to get a sense of the preferred style.	We have included an author summary after the abstract.
8	Early in the Methods section, please state whether the study had a protocol or prespecified analysis plan, and if so attach the document as a supplementary file, referred to in the text. Please highlight analyses that were not prespecified.	This was an exploratory analysis for which there was no prespecified protocol. We have added this for clarification to paragraph 1 of the methods.
9	Where available, please quote p values alongside 95% CI.	We have quoted P values alongside all confidence intervals for odds ratios.
10	Please remove the footnotes (these points can be integrated into the text, we suspect).	We have removed the footnotes and integrated them into the main text in Introduction, paragraph 1, S1 table and S2 table.
11	Throughout the text, please remove the spaces from the square brackets containing reference callouts.	We have removed all spaces preceding the square brackets.
12	Please include a completed checklist for the most appropriate reporting guideline, e.g., STROBE or RECORD, as an attachment with your revision, labelled "S1_RECORD_Checklist" or similar and referred to as such in your Methods section.	We have attached a STROBE checklist labelled S1_Stroke_checklist and referred to table S1 in our methods.
13	In the checklist, please refer to individual items by section (e.g., "Methods") and paragraph number rather than by line or page numbers, as the latter generally change upon publication.	All references in STROBE and in response to Editor and Reviewer comments are now by section rather than line or page numbers.
	Comments	
14	I do, however, have some concerns about the analysis. In addition to points raised by the reviewers, I agree with one of the reviewers that on close inspection, many of the procedures included in Supplementary table 1 are not procedure codes appropriate for bariatric surgery, but include many procedural codes associated with major GI surgery (e.g. gastrectomy for stomach cancer). This raises the issue of the marked weight loss and DM remission being related to coexisting malignancy and the associated weight loss due to the underlying disease or GI surgery.	We used the approach used by Public Health Scotland to identify people who had a history of bariatric surgery which requires the combination of a code for obesity AND the OPCS surgical procedure codes listed in S2 table (personal communication, Public Health Scotland). We have now described how we defined bariatric surgery within the main methods section rather than providing it as a footnote in the supplementary material We have also added a comment on this point to the limitations section.

	The authors would need to provide a detailed explanation to address this concern, which would very much impact on the validity of the analysis.	We agree that some of the procedure codes, are not specific for bariatric surgery. However only 10 of the 488 procedures identified had the least specific codes and we have now modified S2 Table to show the number of procedures for each code within our study population. In addition, we conducted a sensitivity analysis in which we removed people with a code for cancer in the last 5 years and this made little difference to the estimates.<br><br>We hope the explanation provided, along with the sensitivity analysis provides enough information to reassure the reviewer
15	Another major concern, which has not been discussed or addressed, is the potential remission of diabetes that occurs when patients develop significant chronic kidney disease or end stage renal disease, whereby medications are no longer necessary, and A1c may have become normal (or low), due to both diabetes remission as well as co-existing anaemia of chronic disease. A sensitivity analysis to ensure that patients with concomitant malignancy or chronic kidney disease have been excluded would be necessary, in my view	We agree that remission may occur through unintentional weight loss. Interestingly, none of the main consensus statements that define remission of type 2 diabetes specify that weight loss should be intentional. In fact, the 2019 position statement from the Association of British Clinical Diabetologists acknowledges that "weight loss achieved by any means, including unintentional weight loss, may contribute to remission of type 2 diabetes" (page 75 Nagi D, Hambling C, Taylor R. Remission of type 2 diabetes: a position statement from the Association of British Clinical Diabetologists (ABCD) and the Primary Care Diabetes Society (PCDS). Brit J Diab. 2019;19(1):73-6). Therefore, our initial prevalence estimate still fits with the definition of remission. The lack of clarity into the nature of weight loss is probably due to the fact that much of the research into remission of type 2 diabetes has been trial based. However, this observational study has highlighted the importance of this issue for routine practice. We have extended our comment this issue in Discussion, paragraph 6.<br><br>We hope we have clarified that we carried out a sensitivity analysis where we excluded people with malignancy, end stage kidney disease, dementia and liver cirrhosis as this was obviously not clear.<br><br>The point about anaemia of chronic disease is very interesting. Using HbA1c to diagnose type 2 diabetes and remission is imperfect. We have expanded our discussion of the difficulties in using HbA1c to diagnose type 2 diabetes and define its remission and added the reference to our previous publication which discusses this at greater length.

Table 2 Reviewer 1

Point	Comment	Action/Response
	Requests	
16	I found table S3 hard to follow and would appreciate greater explanations as to how to interpret the displayed data.	We have added a sentence to aid general interpretation followed by an example in paragraph 7 of Methods ("statistical analysis")

17	Figure S2 shows the prevalence of remission by age and HbA1c group at diagnosis but the figure header says the results are by duration of type 2 diabetes and age in 2019. I can not see how duration is included in this figure.	This was a typo and has now been corrected.
18	Greater weight loss is associated with remission - did you consider looking at weight change as a percentage of baseline weight? Similarly did change in BMI show any association?	We did also look at change in BMI. This showed similar association as change in weight, however we used change in weight as change in BMI was more difficult to interpret (e.g., change in 1.5kg/m^2 is harder to interpret than a change in 5kg). Using BMI rather than weight increased the proportion of missing data due to missing height values. Given the amount of data already in the table we chose just to present weight change
19	The cut-point of over a year of HbA1c values <48 mmol/mol is valid due to the annual nature of HbA1c measures among people with type 2 diabetes. Did you look at people who failed to meet the criteria because their last HbA1c was just under a year (ie 11 months rather than 12 months)? Would a cut point of at least 10 months change your results?	Our previous paper found that there were 96 unique ways to define remission of type 2 diabetes. We used one year as this was most often used in previous literature describing remission of type 2 diabetes. We investigated the effect of a 10-month cut-off in addition to the 12-month cut off that we used. There were 183 further people when a 10 month cut-point was used instead of 12 months and the prevalence estimate increased by 0.12%. (4.86% remission vs 4.74% remission). We have added this as an additional sensitivity analysis (Results, paragraph 2)
20	Any data on complications - specifically retinopathy around the time of diagnosis which indicates potentially delayed diagnosis?	We agree that this is an interesting question, but we felt it was beyond the scope of this paper to address. We now state that cohort studies are required to investigate associations between remission and complications of diabetes in paragraph 5 of the discussion.
21	You do not find that diabetes duration is associated with risk of remission after adjustment for other covariates. I think this is worthy of some discussion as it seems intuitive that remission is easier in people with new onset diabetes but that is not the case here.	We agree that this is worthy of more discussion and have added more detail in paragraph 4 of the discussion as to why duration was not included. Duration as a continuous variable and as a categorical variable <6 years and $\geq$6 years was not statistically significantly associated with remission in this study. This study used observational rather than trial data and identification of prevalent remission takes a minimum of three readings. As HbA1c is generally measured annually in primary care then diagnosis of remission takes longer to identify compared to trials in which measurements are taken more frequently. We suggest that studies of incidence of remission that include more frequent measurements would be more likely to identify an association between shorter duration of diabetes than we have been able to identify.
22	The association with use of GLT is interesting. I would appreciate some discussion of your interpretation of this - do you think this association is because GLT correlates with sustained hyperglycaemia and symptoms or could it also suggest that treatment with hypoglycaemic agents is deleterious to chances of remission? I appreciate your desire not to overly interpret your findings but I think some discussion is warranted.	We agree that we cannot come to a firm conclusion on whether use of GLT is a causal factor for remission status. However, we have highlighted this association in paragraph 2 of the discussion, not least because it could generate hypotheses to further analyse this association in future research.
23	Can you include some comments on the ethnic make up of your study? It being Scotland I presume it is primarily Caucasian but do you have any estimate on the prevalence of Asian and Black participants?	Ethnicity was missing for 19% of our cohort. We have added ethnicity to the baseline characteristics table: 74.2% of the cohort were white, 18.7% had missing data and 7.1% were other ethnicities. We did not add this to the model due to high proportions of missing and unavailable data and limited numbers of people of non-white ethnicity. We have now added this information to table 1.
24	Have you any data for the length of remission observed in the population. How often do you see a period of an HbA1c <48mmol/mol for at least a year followed by HbA1c values >48mmol/mol. Is the remission you observe likely to be prolonged or more akin to the honeymoon period sometimes observed in early diabetes?	As this is a cross-sectional study, ascertaining the prevalence remission of type 2 diabetes in 2019 was the focus of our research. However, this work informs a baseline cohort that can be followed prospectively to study duration of remission. We agree that this is a very interesting question that warrants further investigation and have indicated that further work is required to describe duration of remission and its implications for risk of complications of diabetes in paragraph 5 of the discussion.

Table 3 Reviewer 2 (Statistical reviewer)

Point	Comment	Action/Response
	Comments	
25	"Factors associated with remission were: older age (OR 1.48 (95% CI 1.34-1.62) for people aged >75 years compared to 45-54 year group), HbA1c <48mmol/mol at diagnosis (OR 1.31 (95%CI 1.24-1.39) compared to 48-52mmol/mol), no previous history of glucose lowering therapy (OR 14.6, 95%CI 13.7-15.5), weight loss from diagnosis to 2019 (OR 4.45 (95%CI 3.89-5.10) for >15kg of weight loss compared to 0-4.9kg weight gain) and previous bariatric surgery (OR 11.9, 95%CI 9.41-15.1)."<br>Did the authors consider investigating interactions between factors?	Two-way interactions between each of age, GLT and<br>HbA1c at diagnosis were investigated, however, none improved overall model fit and therefore were not included these in the final model. We have clarified this in the manuscript in "Methods" paragraph 8.
26	"For the purposes of this analysis we defined remission on the basis of all HbA1c values being <48mmol/mol in the absence of glucose lowering treatment (GLT) for a duration of >365 days before the date of the last recorded HbA1c in 2019, that is on the basis of at least two HbA1c values <48mmol/mol at an interval of at least 365 days. "<br>Did the authors consider undertaking any sensitivity analyses on different definitions of remission?	Many thanks for this, we discussed this at great length, especially in light of our previous systematic review which found over 96 unique definitions of type 2 diabetes remission (Captieux M, Prigge R, Wild S, Guthrie B. Defining remission of type 2 diabetes in research studies: A systematic scoping review. Plos Med. 2020;17(10)). We decided that there was insufficient space to explore the effect of multiple different remission definitions on the prevalence of remission and have now added this as a limitation to the paper, along with describing the effect of using a<br>10 month gap instead of 12 months as requestd by another reviewer
27	"Demographic variables recorded at date of diagnosis of type 2 diabetes were: sex and age (categorised into the following groups: 30 to 44, 45 to 54, 55 to 64, 65 to 74 and 75 and over 150 years)."<br>Can the authors please confirm if age was categorised at the point of data collection, or during data processing? If the latter, did the authors consider including age as a continuous variable in their analyses?	Age was categorised during data processing. We chose to manage the non-linear relationship of age with remission by using the same age categories as the DiRECT trial. We felt that categorising age made the results much easier to interpret to a wider audience and facilitated comparison to DiRECT.
28	"People with no record of an HbA1c >48mmol/mol (6.5%) after diagnosis of diabetes or no HbA1c recorded during 2019 were excluded initially but included for sensitivity analyses for estimates of prevalence of remission."<br>The authors have conducted a valuable sensitivity analysis which helps to demonstrate the robustness in their study findings.	NA
29	The authors have applied comprehensive and rigorous statistical modelling methods, which they describe clearly and concisely. They have appropriately handled missing data by applying MICE, and have suitably conducted a complete case sensitivity analysis. The main study limitations have been thoroughly explored in the discussion section.	NA

Table 4 Review 3

	Comment	Action/Response
	Comments	

30	The finding that previous bariatric surgery was associated with remission of T2D is somewhat confusing. If I understand the methods correctly these patients underwent bariatric surgery before the diagnosis of T2D. What was the time interval between the surgery and diagnosis of T2D? It would seem likely that it was short as it has been shown that new cases of T2D after bariatric surgery is unusual (particularly with modern procedures). Thus, the remission of T2D was not associated with previous bariatric surgery but the effect of the surgery, which is consistent with the effect of bariatric surgery on T2D. Why was not bariatric surgery after the diagnosis of T2D studied?	We have described the way that we identified people with bariatric surgery more clearly in the methods. We used the Public Health Scotland approach for defining bariatric surgery.<br><br>We identified people with an OPCS code for relevant gastro-intestinal surgery at any point prior to 31/12/2019 AND an ICD-10 code for obesity. We did not specific that the bariatric surgery had to follow type 2 diabetes diagnosis but 85% of the study population we identified as having bariatric surgery had the procedure following their type 2 diabetes diagnosis.
31	In addition, the surgical codes presented in Table S1 are confusing. I am not familiar with the OPCS codes but bases on the written description many of the codes used do not represent bariatric surgery, but other procedures performed on the upper gut. Many are procedures done for cancer surgery. Some will have a similar impact on changing the anatomy of the foregut as bariatric surgery with similar impact on gut physiology as bariatric surgery. Yet, they are not bariatric surgery and surgery for cancer comes with other confounding factors.	Please see our response to point 14 above.
32	The authors emphasize that weight loss (achieved without surgery) is an important factor. However, it needs to be pointed out that persistent weight loss without bariatric surgery is difficult to achieve.<br>The findings are also necessary to put in the perspective of impact of remission on hard endpoints. Lessons learned from the look AHEAD study.	Thank you, we have now emphasised the difference between attaining remission and maintaining remission status and quoted the rates of return to clinical diabetes observed in the Look Ahead study after attaining remission after non-bariatric interventions in the discussion, paragraph 6. We have also addressed the lack of evidence that remission or intensive lifestyle interventions have any effect on decreasing cardiovascular disease.

Table 5 reviewer 4

	Comment	Action/Response
	Requests	
33	The authors describe the study aims on Lines 105-107. I would suggest that one of the aims of the study was to identify predictors of T2DM remission in their multivariable regression analysis, in addition to their stated aim of comparing the characteristics of patients with vs. without remission.	This was an exploratory cross-sectional analysis which identified factors associated with remission. We have added a comment that further research is required to develop and validate a prediction model in paragraph 4 of the discussion.
34	Please describe how the decision regarding which covariates to include was derived? If based on existing literature on the predictors of T2DM remission, please cite all relevant papers. Were the covariates selected based on a series of studies that found an association with each of the selected covariates separately or studies that evaluated all the covariates? Or were these covariates decided solely based on clinical rationales? Please describe.	We have described how we chose which covariates to include with relevant references in paragraph 4 of the Methods.
35	Many of the covariates selected are part of the DiaREM score which consists of similar, albeit fewer variables, that have been developed and validated to predict T2DM remission following RYGB surgery (PMID: 24579062, PMID: 28349641, PMID: 26537267). Although this predictive score is limited to remission post bariatric surgery, perhaps the authors can cite this score and/ or any other scores in a more general setting unrelated to bariatric surgery, that consist of a similar set of predictors.	Thank you for this, we have now discussed the similar findings of the DiaRem score in<br>The discussion, paragraph 4. We have highlighted that the variables we found to be associated with remission could be further explored and validated in a future study

36	I'm curious as to why the authors chose to treat age as a categorical variable in its inclusion as a covariate, rather than continuous despite this being available to the authors. Would it not be more optimal to maintain continuous variables as continuous whenever possible?	We discuss the categorisation of age in point 27, in reply to reviewer 2.
37	Why was weight (mentioned in Line 157) mentioned as a covariate? Would BMI, already a covariate, be a more accurate reflection in this case as it accounts for height?	We discuss BMI as a covariate in point 18, in reply to reviewer 1.
38	Did the database contain information about the type of bariatric surgery that patients underwent? Were there any gastric restrictive surgeries (e.g., sleeve gastrectomy or bands) or were they all malabsorptive (e.g., RYGB)? If any details on these are available in the database or perhaps based on local surgical practices during the timeframe of the study (perhaps only certain types of bariatric procedures are offered in Scotland?), it would be great to describe these.	Column 3 in S2 Table provides information about the type of surgery the patients underwent. We have additionally added a column in S2 table to show the number of people in our cohort with each OPCS code
39	Figure 1 - This figure should be mentioned in the first part of the Results section when the included population is first described, rather than in the Methods section.	The reference to figure 1 has now been removed from the methods section.
40	Figure 3 - Very helpful forest plot. I would suggest adding labels to the x-axis for easier interpretation for readers.	X- axis labels are on the right side of the x-axis for all forest plots including figure 3, Figure S5 and Figure S6.
41	Was bariatric surgery associated with higher SES? Perhaps this association is strong enough to account for the fact that SES was not a predictor in the multivariable analysis (once SES was factored in)?	We investigated the association between bariatric surgery and SIMD to address this comment. Adding bariatric surgery as a covariate to the univariable model of SIMD quintile and remission had a negligible effect on the association between SIMD quintile and remission suggesting that the association between bariatric surgery and SES is not strong enough to account for SES not being a predictor of remission in the multivariable model
42	Please briefly describe the DiRECT trial that is discussed in the final paragraph of the Results section.	We have added a section to summarise the DiRECT trial in the introduction, paragraph 2.
43	It seems that bariatric surgery was a predictor of T2DM remission independent of weight loss since both were statistically significant predictors in the multivariable model. If that's the case, this is an interesting finding that may be worth explicitly mentioning in the Results & discussing further in the Discussion section.	We have highlighted the independent association between bariatric surgery and remission and weight loss and remission in Results, paragraph 4 and Discussion, paragraph 2.
44	Minor comment - Lines 310-311 - This statement "... inversely associated with socioeconomic deprivation..." suggests that remission was associated with higher SES but in its current form describing the inverse relationship with socioeconomic deprivation, the statement is somewhat difficult to understand and could be simplified.	We have simplified this sentence in Discussion paragraph 2.
